# Influence of Preoperative Chemoradiotherapy on the Surgical Strategy According to the Clinical T Stage of Patients With Rectal Cancer

**DOI:** 10.1097/MD.0000000000002377

**Published:** 2015-12-31

**Authors:** In Ja Park, Jong Lyul Lee, Yong Sik Yoon, Chan Wook Kim, Seok-Byung Lim, Jong Seok Lee, Seong Ho Park, Jin Hong Park, Jong Hoon Kim, Chang Sik Yu, Jin Cheon Kim

**Affiliations:** From the Department of Colon and Rectal Surgery (IJP, JLL, YSY, CWK, S-BL, JCK); Department of Radiology (JSL, SHP); and Department of Radiation Oncology (JHP, JHK), University of Ulsan College of Medicine and Asan Medical Center, Seoul, South Korea.

## Abstract

The aim of this study was to evaluate the pathologic responses and changes to surgical strategies following preoperative chemoradiotherapy (PCRT) in rectal cancer patients according to their clinical T stage (cT).

The use of PCRT has recently been extended to less advanced disease.

The authors enrolled 650 patients with cT2 to 4 mid and low rectal cancer who received both PCRT and surgical resection. The rate of total regression and the proportion of local excision were compared according to the cT category. The 3-year recurrence-free survival (RFS) rate was compared using the log-rank test according to patient cT category, pathologic stage, and type of surgical treatment.

Patients with cT2 were older (*P* = 0.001), predominately female (*P *= 0.028), and had low-lying rectal cancer (*P* = 0.008). Pathologic total regression was achieved most frequently in cT2 patients (54% of cT2 versus 17.6% of cT3 versus 8.2% of cT4; *P* < 0.001). Local excision was performed on 42 cT2 (42%) and 24 cT3 (5.2%) patients (*P *< 0.001). The 3-year RFS rates differed according to both cT (*P* < 0.001) and ypT stage (*P* < 0.001). Among patients with ypT0 to 1 disease, the 3-year RFS did not differ according to the type of surgical treatment received (*P *= 0.5).

Total regression of the primary tumor and a change in the surgical strategy after PCRT are most commonly seen in cT2 disease. Although PCRT is not generally indicated for cT2 rectal cancer, optimal surgical treatment may be achieved with the tailored use of PCRT.

## INTRODUCTION

The current standard treatment for locally advanced rectal cancer is preoperative chemoradiotherapy (PCRT), which has been shown to successfully downstage tumors and promote favorable clinical outcomes.^1,2^ The benefits of PCRT are optimal in patients with tumors that are highly responsive to this treatment and in patients who have certain clinical characteristics that are associated with superior treatment outcomes.^3–5^ Generally, PCRT has been indicated only for advanced rectal cancer [clinically diagnosed as T3 to 4 (cT3 to 4)] and rectal cancer presenting with metastatic lymph nodes (LNs). Recent studies, however, have reported treatment of cT2 rectal cancer using PCRT.^6–8^ The standard of treatment for most stage I rectal cancers is surgery alone, specifically total mesorectal excision (TME).^9^ Local excision (LE), including transanal excision and transanal endoscopic microsurgery, has been explored as a surgical treatment for stage I disease because of the morbidity and/or functional derangement associated with TME. Local excision alone, however, demonstrates inferior oncologic outcomes in comparison with TME.^10,11^

Despite continuing reports on inferior outcomes following LE, the rate of LE use to treat stage I tumors has steadily increased.^12^ The continued use of LE to treat stage I tumors may be because of the lower morbidity rates and better long-term functional outcomes associated with LE compared with TME. For distal rectal cancers in particular, LE offers the promise of sphincter preservation, whereas TME often results in permanent ostomy creation. The use of PCRT in cT2 disease is expected to improve oncologic outcomes comparison with LE, therefore extending the indications of LE to cT2 disease for functional improvement. The influence of surgical strategies might be closely associated with the rate of total regression (TR) of primary rectal tumors because the tumor response to CRT has emerged as an important predictor of tumor control and patient survival.^13,14^

We evaluated the rate of TR for primary tumors following PCRT and the influence of PCRT on surgical strategies (ie, the rate of LE according to cT category) in patients with mid to low rectal cancer. In addition, we evaluated oncologic outcomes in a series of cT2 rectal patients according to surgical treatment (LE versus TME).

## METHODS

### Patients, Diagnosis, and Clinical Staging

We included 650 patients with primary mid to low rectal cancer (located within 10 cm of the anal verge) that received treatment with PCRT followed by surgical resection (including LE) between January 2011 and December 2013 at Asan Medical Center, Seoul, South Korea. Patients with simultaneous distant metastases on pretreatment work-ups or with a prior or concurrent malignancy were excluded. Patients who did not receive any kind of surgical treatment or who were diagnosed using techniques other than magnetic resonance imaging (MRI) were also excluded. The clinical stage of each tumor was diagnosed on MRI using a high spatial resolution phased-array Imagnetic resonance imaging technique. An MRI diagnosis of a cT3 lesion was based on the presence of tumor signal intensity that extended through the muscle layers into the perirectal fat with a broad-based bulging configuration and continuity with the intramural portion of the tumor. Tumors located within the muscle layer were diagnosed as cT3 lesions. Tumor signal intensity that extended beyond the perirectal fat or demonstrated a loss of plane between the adjacent organs was diagnosed as a cT4 lesion. Metastatic status of the LNs was ascertained by considering nodal size and morphologic characteristics, such as signal intensity, border, contour, shape, and texture. This practice is in contrast to the method agreed on and practiced by many experts in which a single criterion, such as a size threshold, is evaluated.^15^

### Preoperative Chemoradiotherapy, Surgical Treatment, and Pathologic Examination

The PCRT regimen consisted of a 45-Gy dose of pelvic external beam radiation delivered in 25 fractions during 5 weeks. During the last week of treatment, patients received a 5.4-Gy boost to the primary tumor delivered in 5 (second daily) fractions, cumulating in a total radiation dose of 50.4 Gy. Chemotherapy was delivered as 2 cycles via an intravenous bolus of 5-fluorouracil (FU) (375 mg/m^2^/d) and leucovorin (20 mg/m^2^/d) for 3 days during the first and fifth weeks of radiation therapy or as oral capecitabine (1650 mg/m^2^/d), administered twice-daily during radiation therapy. Approximately 4 weeks after completing PCRT, clinical stage was reevaluated using pelvic MRI, abdominopelvic computed tomography, and sigmoidoscopy. Surgical resection was planned within 6 to 8 weeks of PCRT completion. Patients were supposed to undergo radical resection according to the principles of tumor-specific mesorectal excision. Patient refusal of radical surgery and poor performance status were reasons for undergoing LE instead of TME following PCRT. Patients who chose LE were fully informed about the tumor response to PCRT and the surgical options between radical resection and LE. Each patient provided written informed consent before treatment.

Adjuvant chemotherapy was recommended for all medically fit patients who received PCRT and radical resection and consisted of infused 5–FU or capecitabine for 6 months. Oxaliplatin-based chemotherapy was administered to some patients based on their postoperative pathologic results.

### Pathologic Examination, Follow-up, and Oncologic Outcomes

Dedicated gastrointestinal cancer pathologists performed standard pathologic tumor staging. Tumors were pathologically staged according to the guidelines of the American Joint Committee on Cancer (7th edition). The LNs were identified by manual dissection of mesorectum and examined using 1 to 3 separate sections per node. Pathologic responses to PCRT were evaluated in the resected specimens using the tumor regression grade system suggested by the Gastrointestinal Pathology Study Group of the Korean Society of Pathologists.^16^ Tumor regression was scored as follows; TR with no residual tumor cells and only fibrotic mass, near-total regression with microscopic residual tumor in the fibrotic tissue, and moderate regression with easy-to-find irradiation-related changes with residual tumor; minimal regression with a dominant tumor mass with obvious irradiation-related changes, or no regression or evidence of irradiation-related changes, such as fibrosis, necrosis, or vascular changes.

Postoperative follow-up consisted of routine physical examinations and carcinoembryonic antigen measurements every 3 to 6 months, along with abdominal pelvis and chest computed tomography every 6 months to 1 year. Colonoscopies were performed at 6 months or 1-year postoperatively and every 2 to 3 years thereafter. Recurrence-free survival (RFS) was defined as the time between surgery and the first recurrence event or death.

### Statistical Analysis

Pearson χ^2^ test, Fisher exact test, or Student *t* test were used for comparison of clinicopathologic characteristic of the patients according to their cT category as applicable. The associations between surgical treatment and pathologic results were also compared between patient groups. Cases with disease recurrence or death from any cause were identified as failures at the time of recurrence or death for RFS analysis. Noncancer deaths were not censored. The 3-year RFS rates were determined using the Kaplan–Meier method, and compared using the log-rank test between groups. Cox proportional hazards regression analysis was used to perform the multivariate comparisons. In all analyses, *P* < 0.05 was considered statistically significant. All statistical analyses were performed using SPSS version 21.0 (IBM Statistics, Armonk, NY).

## RESULTS

### Patient Characteristics

We included 650 patients who met inclusion criteria. The median age was 61 years [interquartile range (IQR) = 48–66 years]. Men (64.6%) were predominant among patients. The median distance of the tumor from the anal verge was 6 cm (IQR = 4–8 cm). Most tumors were cT3 on preoperative staging. Concurrent chemotherapy using of 5-FU were used in 42.4% and capecitabine in 57.6% of the patients. Sixty-seven patients underwent LE, and the remainder underwent TME or tumor-specific mesorectal excision depending on extent and location of the tumor. Sphincter-preserving operations were performed on 81.5% of the patients treated with radical resection. In total, 143 patients (22%) demonstrated TR on tumor regression grade.

### Clinicopathologic Characteristics and Surgical Treatment According to Clinical T Stage Category

The cT2 group was older (*P* = 0.001), predominately female (*P* = 0.028), and demonstrated low-lying rectal cancer (*P* = 0.008). Patients with a lower cT category were also more likely to demonstrate TR of the primary tumor following PCRT (*P* < 0.001). Our analyses showed that 54% of patients with cT2 disease demonstrated TR but only 8.2% of patients with cT4 disease achieved TR of the primary tumor. The pathologic T stage varied according to cT category. Although tumors in patients in the cT2 group demonstrated downstaging to a ypT0 to 1 primary tumor in 79 cases (79%), tumors in patients in the cT3 group were downstaged to a ypT0 to 2 primary tumor in 250 cases (52.8%). A total of 73% of patients with cT4 disease, however, maintained a ypT3 to 4 disease status. Among the patients who underwent radical resection, the ypN+ rate also differed according to cT category. Among patients with cT2 disease, only 1 patient demonstrated a ypN+ tumor (1.7%). The number of ypN+ tumors increased according to the ypT stage. ypN+ tumors accounted for 7 of the 144 ypT0 to 1 tumors (4.9%) that underwent radical resection. Among patients with ypT2 disease, the ypN+ rate was 12.4% but abruptly increased to 39% and 70% in patients with ypT3 and ypT4 disease, respectively.

Local excision was more frequently performed on cT2 cases (42%). Sphincter preservation, including LE, was most commonly performed for cT2 disease, although the cT2 group demonstrated more low-lying rectal cancer cases. Among patients with cT3 disease, only 5.2% of cases underwent LE, although 17.6% of these patients demonstrated TR of the primary tumor. No patient with cT4 disease underwent LE. The surgical strategy was changed from the current standard surgical treatment to an alternative method in 77.8% of cT2 and 29.3% of cT3 patients with TR (Table 1).

**TABLE 1:**
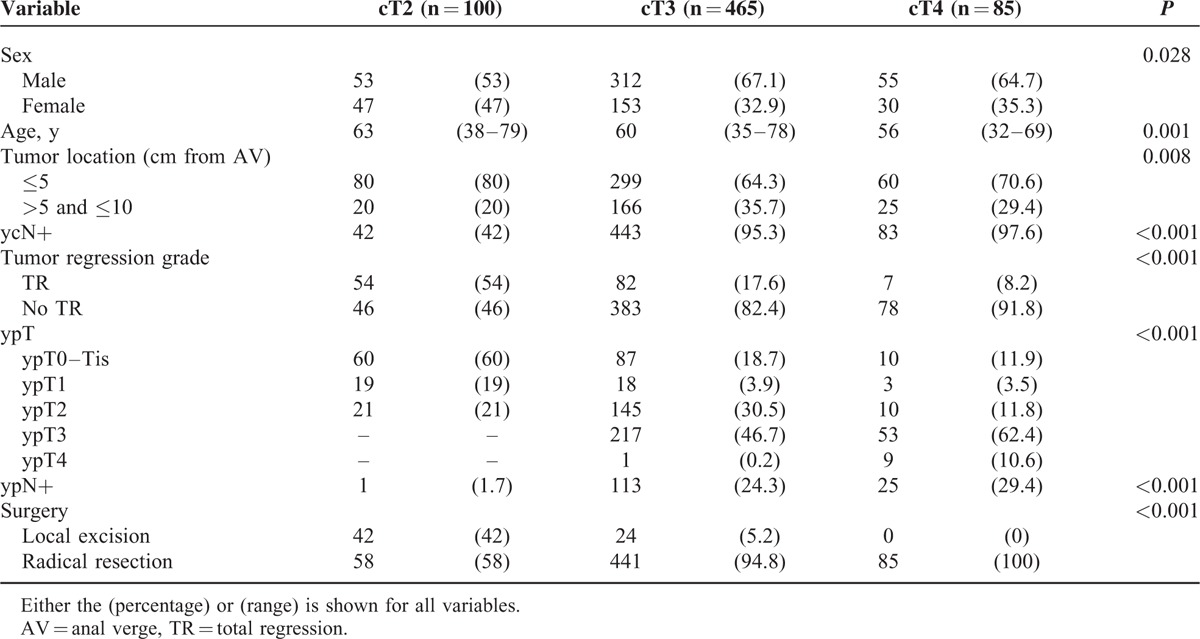
Clinicopathological Characteristics of the Study Patients According to Their Clinical T Stage Category

### Accuracy of Pelvic Magnetic Resonance Imaging for Predicting the ypT Stage Following Preoperative Chemoradiotherapy According to the Clinical T Stage Category

For 323 patients, pelvic MRI following PCRT accurately predicted the ypT stage. Underestimation of ypT stage occurred in 8.2% of patients. The accuracy of post-PCRT MRI, in terms of predicting TR of the primary tumor, differed according to cT category. Among patients with cT2 disease, TR of the primary tumor was predicted in 39.8% of patients, but it was only predicted in 10% of patients with either cT3 or cT4 disease. The accuracy of post-PCRT MRI also differed according to ypT stage. Magnetic resonance imaging could not accurately predict ypT stage in approximately 40% of patients with ypT0 to 2 disease, but this inaccuracy increased to 77.8% among patients with ypT3 disease (Table 2).

**TABLE 2:**
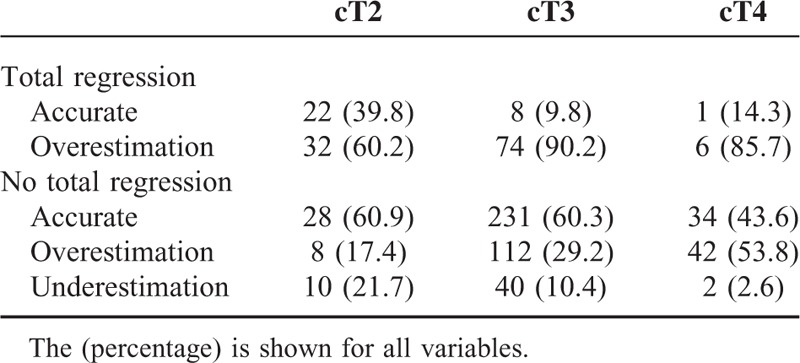
Use of Magnetic Resonance Imaging to Predict Primary Tumor Regression in the Rectal Cancer Patients According to the Clinical T Stage Category After Preoperative Chemoradiotherapy

### Recurrence and Survival

**FIGURE 1:**
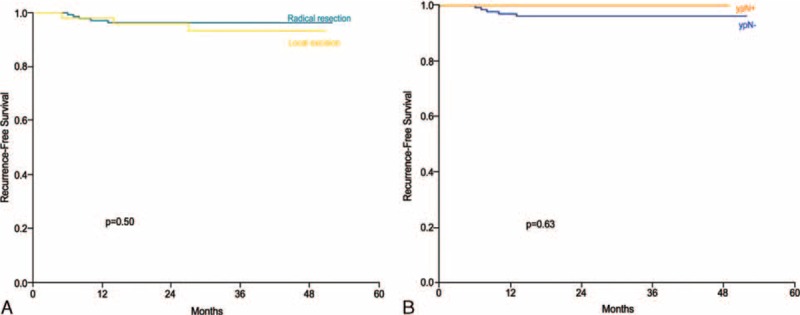
The 3-year recurrence-free survival rates significantly differ according to the (A) clinical T stage category and (B) ypT stage of rectal cancer patients.

**FIGURE 2:**
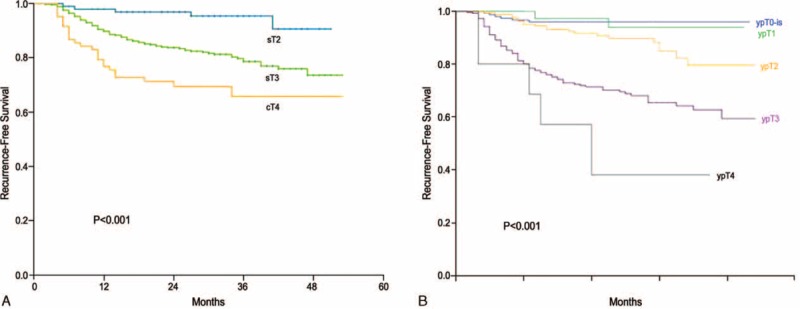
The 3-year recurrence-free survival rates in rectal cancer patients with ypT0 to 1 disease do not differ according to (A) the type of surgical resection or (B) the metastatic lymph node status.

The median follow-up period was 30 months (IQR = 21–39 months) for the entire study cohort and did not differ according to cT category. Overall, recurrence was observed in 115 patients (17.7%). Nine patients demonstrated only local recurrence, 98 patients demonstrated only systemic recurrence, and 8 patients demonstrated both local and systemic recurrence. The lung was the most common initial metastatic site (68 of 115 patients; 59.1%). For the entire cohort, the RFS at 3 years was 79.6%. The 3-year RFS differed according to cT category and ypT stage (Fig. 1). Among patients with ypT0 to 1 disease, the 3-year RFS did not differ according to the type of surgical resection or LN metastasis (Fig. 2). According to the adjusted multivariate Cox regression analysis, the type of surgical resection, sex, age, location, and cT category were not associated with RFS among patients with ypT0 to 1 disease (Table 3).

**TABLE 3:**
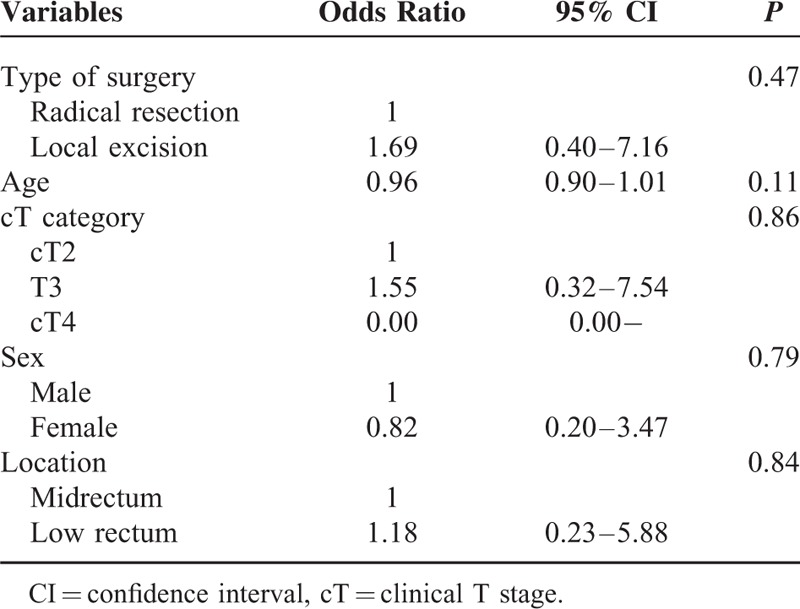
Multivariate Analysis of the Factors Associated with 3-Year Recurrence-free Survival

## DISCUSSION

Our current study findings indicate that PCRT can result in significantly higher TR of the primary tumor in rectal cancer patients and provide a greater influence on subsequent surgical treatments for cT2 disease than for cT3 to 4 diseases, which are the current standard indications for PCRT. It is unclear if the rate of TR would increase, if PCRT were administered to patients with low-cT disease. Many studies on administering PCRT to treat cT2 disease report discrepant results.^6–8^ Some studies report a TR of approximately 20% for cT2 disease, similar to locally advanced rectal cancer, whereas others report a TR of close to 50% for primary rectal tumors.^8,17–19^ Most previous studies are limited by the small number of enrolled patients, and the higher rate of ypT0 in these reports may be influenced by a potential selection bias caused by including patients who intentionally planned to undergo LE after PCRT and were usually diagnosed with less invasive tumor characteristics. In contrast, patients with cT3 to 4 disease typically had bulky and invasive tumors, which make clear resection margins difficult, and the expected effects of PCRT differed slightly in these patients relative to those with cT2 disease. Regardless of these possible biases, some randomized trials on administering PCRT for cT2 disease^6,7^ reported higher rates of TR for primary tumors compared with cT3 to 4 disease (usually 15%–20%).^4,20^ Therefore, it is hard to determine whether a low-cT category would more frequently result in TR of the primary tumor.

Here, we report TR of primary tumors in 52.9% of patients. This relatively high TR rate may have been caused by selective PCRT administration for cT2 disease at our institution or inaccurate clinical staging. When selecting patients for PCRT, the accuracy of the imaging modality is key.^21,22^ Patients with ≤cT1 disease, which can be overdiagnosed as cT2 disease, may have been included in our current cohort and could have influenced primary tumor regression and subsequent surgical treatment results. Alternatively, it may be that TR is achieved more frequently in patients with low-cT disease.

Downstaging of primary tumors broadens the surgical treatment options following PCRT. Patients with low-lying rectal cancer who are not supposed to undergo sphincter-preserving surgery or who have profound functional derangement after sphincter preservation in particular must undergo LE after PCRT as an alternative approach to preserve the sphincter and anorectal functions.^23,24^ Even though LE has demonstrated comparable oncologic outcomes to TME for cT2 rectal cancer following PCRT in randomized trials,^6,7^ in practice this procedure is not indicated for all cT2 rectal cancer patients. Surgical treatment is determined according to the extent of tumor regression following PCRT.

In our current study, 17.8% of the cT3 rectal cancer patients demonstrated TR of their primary tumor, but only 5.2% of these patients underwent LE. In contrast, 54% of cT2 rectal cancer patients demonstrated TR, and 42% of these cases underwent LE. No patients with cT4 rectal cancer underwent LE. In patients with cT2 rectal cancer, PCRT was usually administered with the intention of performing LE, and this selection bias may have resulted in the observed high TR rate. A selection bias would also influence the accuracy of clinical restaging by MRI following PCRT.

Accurate clinical restaging following PCRT is critical for implementing LE. We found that the accuracy of post-PCRT MRI for ypT staging differed according to the ypT stage. Disease at a stage earlier than ypT2 is hard to accurately predict, but we could predict 77.8% of ypT3 disease in patients using post-PCRT MRI. The accuracy of MRI for ypT staging also differs depending on the cT stage. Total regression prediction was correct in 21.7% of all of our patients with TR. This, however, differed according to the cT category, and although TR was predicted in 39.8% of cT2 patients, this rate decreased to 9.8% and 14.3% in patients with cT3 and cT4 disease, respectively. This result suggests that restaging following PCRT may be influenced by the clinical stage before PCRT and by intentions regarding subsequent treatment. This inconsistency between clinical TR on MRI and pathologic TR may also influence the choice of surgical treatment. Similar discrepancies between clinical and pathologic TR have been reported in previous studies.^3–5^

The major concern in performing LE for rectal cancer following PCRT is inadequate treatment of regional LNs, which may become involved by the tumor.^25,26^ The rate of LN metastasis increases with advanced ypT stage. Reportedly, 0% to 15% of patients with ypT0 disease following PCRT have metastatic LNs.^27–29^ In our current study, pathologically diagnosed LN metastasis was 1.7% in the cT2 group and 4.9% among patients with ypT0 to 1 disease. By accurately predicting the ypT stage, we can predict the risk of LN metastasis.

The oncologic importance of metastatic LNs may differ by ypT stage. As with the paradoxical oncologic outcomes between stage IIB and IIIA colon cancer,^30^ the impact of metastatic LNs on oncologic outcomes might differ depending on the depth of tumor invasion. The 3-year RFS rate did not differ according to the ypN stage in patients with ypT0 to 1 disease, but patients with ypN+ cancer did demonstrate a poorer 3-year RFS rate in the ypT2 to 4 stage. In the ypT0 to 1 patients, the type of surgical treatment (LE versus TME) was not associated with RFS either. If the oncologic impact of metastatic LNs does differ according to depth of tumor invasion, administration of LE after PCRT could be extended to patients who have metastatic LNs with less oncologic impact. More concrete evidence, however, is required to clarify the oncologic influence of metastatic LNs in terms of the depth of tumor invasion after PCRT.

Our study had several limitations inherent to its retrospective design. First, the selection of patients for PCRT in our study series, especially those with cT2 disease, was based on the expectation of changes in surgical strategy; this expectation could have influenced the surgical treatment selection and TR rate after PCRT. In comparison with previous studies, the TR rate among our patients with cT2 disease was higher, which could have been caused by selection bias or the limitations of pre-PCRT MRI. Like others, we found that overestimation is more common than proper staging in patients with ypT0 disease. If patients with <cT2 disease were included in the cT2 group, this would have incorrectly increased the prevalence of ypT0 to 1 disease. In addition, the clinical information for the included patients was open to the interpretation of the radiologist, and this could have influenced the MRI-based restaging that was performed following PCRT in our study patients. In addition, we found that oncologic outcomes did not differ according to surgical treatment or ypN stage among patients with ypT0 to 1 disease. Considering the possibility of late recurrence after PCRT, a longer follow-up period is needed to ascertain the association between these factors and oncologic outcomes. Regardless of these limitations, we could successfully analyze current practice patterns and suggest careful approaches for rectal cancer patients who are treated with PCRT to correctly determine the clinical stage of the disease and appropriate surgical treatments.

In conclusion, primary tumor regression is more frequent in rectal cancer patients with cT2 disease. Preoperative chemoradiotherapy would significantly influence the surgical strategy for patients with cT2-stage disease, as they demonstrate higher TR of the primary tumor than patients with cT3 or cT4 disease. Future studies should include a larger patient population and longer-term follow-up periods to confirm our observed oncological outcomes according to the surgical treatment administered to select rectal cancer patients.
